# Enhancing Pearl oyster mushroom (*Pleurotus ostreatus* (Jacq.) P. Kumm) performance by evaluating the influence of potassium humate and wheat straw on yield and biochemical attributes

**DOI:** 10.1186/s12870-025-06988-8

**Published:** 2025-07-19

**Authors:** Sayed Hussein Abdelgalil, Esraa Mohamed, Islam I. Teiba, Sobhi F. Lamlom, Ahmed M. Abdelghany, Mohamed E. Shalaby

**Affiliations:** 1 Central Laboratory of Organic Agriculture (CLOA), Agricultural Research Center (ARC), Giza, Egypt; 2 Horticulture Research Institute, Vegetable Cross Pollination Dep, Agricultural Research Center (ARC), Giza, Egypt; 3 Botany Department, Faculty of Agriculture, Tanta University, Tanta City, 31527 Egypt; 4 Plant Production Department, Faculty of Agriculture Saba Basha, Alexandria University, Alexandria, 21531 Egypt; 5 Crop Science Department, Faculty of Agriculture, Damanhur University, Damanhur, 22516 Egypt

**Keywords:** *Pleurotus ostreatus*, Substrate amendment, Growth optimization, Potassium humate, Bioactive compounds

## Abstract

This study investigated the effects of various combinations of potassium humate and raw wheat straw (RWS) treatments on the growth, yield, and quality of *Pleurotus ostreatus* (Jacq.) P. Kumm across two growing seasons. A total of seven treatment combinations (H0_RWS, H0.25_RWS, H0.5_RWS, H0.75_RWS, H1_RWS, H1.25_RWS, and H1.5_RWS) were applied to evaluate eleven traits, including soluble sugar content (SSC), fruiting body yield (FBY), biological efficiency (BE), and biochemical attributes. The analysis of variance revealed significant effects of both season and treatment on all traits, with notable interactions. The H0.5_RWS treatment demonstrated the highest improvements, increasing SSC by 15.3%, FBY by 18.6%, and vitamin C (VC) by 12.4% compared to the control. Utilizing principal component analysis (PCA) showed that the first two components explained 89.5% of the variance, with strong associations between SSC, VC, FBY, total phenolic content (TPC), BE, and peroxidase activity (POD) on PC1. The H0.5_RWS treatment was identified as the most effective for enhancing these parameters. Heatmap and radar plot analyses further confirmed the positive influence of moderate potassium humate doses, while higher doses resulted in reduced benefits. Correlation analysis showed strong positive relationships between yield and both total sugars (R² = 0.99) and BE (R² = 0.97). The results emphasize the substantial importance of balanced potassium humate application in enhancing the production and nutritional quality of *P. ostreatus*, providing valuable insights to optimize the cultivation practices of this species.

## Introduction

The global demand for sustainable food production has intensified interest in alternative protein sources, with edible fungi emerging as a promising solution. Among these, the oyster mushroom (*Pleurotus spp*.) represents one of the most economically significant cultivated fungi, ranking second in worldwide production after *Agaricus bisporus* [1]. Additionally, it is rich in bioactive compounds such as polysaccharides and terpenoids, which confer a wide range of health benefits, including anti-cancer properties, anti-inflammatory effects, antibacterial activity, blood sugar regulation, cholesterol reduction, and lowering of blood pressure [2, 3]. These nutritionally rich and medicinally potent mushrooms are also capable of decomposing various lignocellulosic materials. Thus, their cultivation not only provides a protein-dense food source but also contributes to reducing environmental pollution [4, 5].

The cultivation of oyster mushrooms offers additional environmental benefits through their unique ability to decompose various lignocellulosic materials, effectively converting agricultural waste into valuable biomass while simultaneously reducing environmental pollution [6–9]. This bioconversion capacity has made oyster mushroom cultivation particularly attractive to farmers seeking to diversify their income streams while addressing waste management challenges. Currently, three primary cultivation approaches are employed: raw material cultivation, sterilized cultivation, and compost-based cultivation, each offering distinct advantages in terms of yield optimization, contamination control, and resource efficiency [2, 10–12]. The substrate composition plays a crucial role in determining mushroom yield and quality, with organic waste materials such as rice and wheat straws, sugarcane bagasse, sawdust, and various agricultural residues serving as cost-effective cultivation media [13, 14]. Among these substrates, wheat straw has consistently demonstrated superior performance in supporting *P. ostreatus* growth, yield, and biochemical attribute development compared to alternative materials [13]. However, the potential for substrate enhancement through targeted amendments remains an active area of research, particularly in the context of optimizing both quantitative and qualitative mushroom parameters.

Potassium humate, a naturally occurring organic compound derived from plant material decomposition, has emerged as a promising substrate amendment in agricultural systems. This humic substance has demonstrated significant benefits across various crops, enhancing yield, stress tolerance, and nutritional quality through its role in nutrient availability, soil structure improvement, and plant physiological processes [15–18]. Recent studies have shown that potassium humate application significantly improves photosynthetic performance and agronomic traits in foxtail millet [19], enhances stress tolerance and antioxidant defense systems in rice under arsenic stress [20], mitigates water deficit stress in peanut through proline regulation and antioxidant enhancement [21], and improves wheat growth under saline conditions [22]. The compound’s effectiveness stems from its influence on critical plant functions including enzyme activation, osmoregulation, and protein synthesis, making it a valuable tool for crop optimization across diverse agricultural systems [15]. Despite these well-documented benefits in conventional agriculture, the application of potassium humate in mushroom cultivation remains largely unexplored, representing a significant knowledge gap in fungal production systems. The limited research available on humic substances in mushroom cultivation has shown promising preliminary results, with studies on various Pleurotus species indicating potential benefits for growth parameters and nutritional composition [23, 24]. However, these investigations have been constrained by narrow concentration ranges, single-season evaluations, and limited biochemical characterization, leaving critical questions unanswered regarding optimal application rates and comprehensive effects on mushroom quality attributes [25]. Furthermore, the specific interaction between potassium humate and wheat straw substrate has not been systematically investigated, despite the recognized superiority of wheat straw as a cultivation medium [25].

The current literature lacks comprehensive studies that systematically evaluate potassium humate concentrations in mushroom cultivation while simultaneously assessing yield, nutritional quality, and biochemical attributes across multiple growing seasons. Existing studies have focused primarily on single parameters or limited concentration ranges, failing to establish clear dose-response relationships or identify optimal application strategies. Additionally, the integration of advanced multivariate statistical approaches for treatment optimization in mushroom cultivation remains underutilized, representing a missed opportunity for data-driven cultivation improvements. Based on the documented benefits of potassium humate in other agricultural systems and the limited evidence from mushroom studies, we hypothesize that moderate concentrations of potassium humate (0.5–0.75%) will significantly enhance P. ostreatus yield, biological efficiency, and nutritional quality when combined with wheat straw substrate, while higher concentrations may exhibit diminishing returns or inhibitory effects due to potential osmotic stress or nutrient imbalance. Given the established benefits of potassium humate in agricultural systems and the lack of comprehensive research in mushroom cultivation, this study addresses the fundamental question: What is the optimal concentration of potassium humate when combined with wheat straw substrate to maximize yield, nutritional quality, and biochemical attributes of P. ostreatus?

To address this question, the present study aimed to: (1) evaluate the effects of varying potassium humate concentrations (0.25–1.5%) combined with wheat straw on P. ostreatus growth, yield, and quality parameters across two growing seasons; (2) identify optimal treatment combinations through comprehensive biochemical profiling including soluble sugars, vitamin C, phenolic compounds, and antioxidant activities; (3) establish concentration-response relationships using advanced multivariate statistical analysis including principal component analysis, heatmap visualization, and correlation analysis; and (4) provide evidence-based recommendations for commercial oyster mushroom cultivation optimization.

## Materials and methods

### Experimental design and treatments

A commercial strain of oyster mushroom white tree *Pleurotus ostreatus* (Jacq.) P. Kumm were taken in the form of commercial fruiting body packages from Agriculture Research Center, Giza, Egypt. A commercial strain of oyster mushroom white tree (*P. ostreatus*) cultured on potato dextrose agar (PDA) (PanReac AppliChem, Spain) media at the Central Laboratory of Organic Agriculture (CLOA), Agricultural Research Center (ARC), Egypt. The experiment was conducted using a completely randomized design (CRD) during the 2022 and 2023 growing seasons, with three replications per treatment. The key environmental differences between seasons were shown in table S1. The objective was to evaluate the effects of varying levels of potassium humate on the production of *Pleurotus ostreatus* (Jacq.) P. Kumm. Seven distinct treatments (Table 1) were tested, each with nine replicates, to assess their impact on fruiting body yield and other related traits.

**Table 1:** Raw wheat straw and potassium humate treatments for *P. ostreatus* growth

No.	Treatment	Treatment ID
T1	500 g of raw wheat straw (RWS) without potassium humate	H0_RWS
T2	500 g of RWS with 0.25 g of potassium humate	H0.25_RWS
T3	500 g of RWS with 0.5 g of potassium humate	H0.5_RWS
T4	500 g of RWS with 0.75 g of potassium humate	H0.75_RWS
T5	500 g of RWS with 1 g of potassium humate	H1_RWS
T6	500 g of RWS with 1.25 g of potassium humate	H1.25_RWS
T7	500 g of RWS with 1.5 g of potassium humate	H1.50_RWS

Potassium humate (commercial grade, containing 10–12% K₂O, 65–70% humic acid, 15–20% fulvic acid, pH 9–11, > 95% water solubility) was sourced from Nano Chem Company, Egypt. The potassium humate was dissolved in distilled water (1:10 w/v ratio) to ensure complete solubility and then thoroughly mixed with the moistened raw wheat straw substrate before inoculation with mushroom spawn. Special care was taken to ensure uniform distribution and homogeneous mixing of the potassium humate solution throughout the substrate to prevent localized concentration variations that could affect mushroom development. Potassium humate was thoroughly mixed with the moistened raw straw substrate before inoculation with mushroom spawn. Each treatment was applied to nine bags per replicate, ensuring uniform distribution and mixing of the potassium humate with the substrate. The experimental conditions were maintained consistently across all treatments, including temperature, humidity, and light exposure, to ensure reliable and comparable results.

### Preparation of straw and spawn incubation

Raw wheat straw (RWS) was initially moistened by soaking it overnight in water. Following the soaking process, the straw was sterilized using an autoclave at 121 °C for 30 min to eliminate any potential contaminants [4]. After sterilization, the straw was allowed to cool to room temperature. Excess water was carefully drained off to achieve the desired moisture content for optimal mushroom growth. Once prepared, the moistened and sterilized substrate was manually packed into clear polyethylene bags measuring 20 × 40 cm, each containing 1 kg of the substrate. The bags were then sealed to maintain substrate moisture and prevent contamination. Mushroom spawn was introduced to the substrate at a rate of 5% of the wet mass of the substrate [26]. This inoculation rate ensured effective colonization of the substrate by the mushroom mycelium. After inoculation, the bags were incubated under controlled environmental conditions—maintaining consistent temperature, humidity, and light exposure—to support mycelial growth and subsequent fruiting body development.

### Culture conditions for spawn growth and fruiting body development

The inoculated substrate was incubated at 24–28 °C during the mycelium running phase, with adequate light and ventilation provided to facilitate colonization until the substrate was fully covered with mycelium [4]. Once colonization was complete, the mushroom bags were transferred to a fruiting chamber. Portions of the polyethylene bags were cut to create openings, which allowed the mushrooms to develop pinheads more effectively. Fruiting bodies were harvested approximately one week after pinhead formation.

### Data recording

#### Growth performance and yield assessment

Data recorded included the spawn running phase, total fruiting body yield (across all flushes, g kg^−1^) of moistened substrate, spent substrate weight (g), and biological efficiency (BE) in percentage. Biological efficiency was calculated using the formula: BE (%) = (weight of fresh mushroom fruiting bodies/weight of dry substrate) × 100 [4].

Mushrooms (10 g) were added to 100 mL of distilled water and heated for 30 min. The resulting mixture was filtered through qualitative Whatman_ No.1 paper, and the resulting filtrate was transferred into 50-mL volumetric flasks and diluted with distilled water until the volumetric flask was full.

#### Determination of total phenolics, soluble solids, and sugar contents

Total phenolic content of the aqueous extracts was determined according to the method described by [27], with modification. One mL of a sample was mixed with 1.0 mL of Folin-Ciocalteu’s phenol reagent. After 3 min, 1.0 mL of 35% saturated sodium carbonate was added, and the mixture was made up to 10 mL using addition of deionized water. The mixture was kept for 90 min at room temperature in the dark. The absorbance was measured using UV-Vis spectrophotometer at 725 nm against a blank. Gallic acid was used as a reference standard. The total phenolic content was expressed as mg of gallic acid equivalents (GAE) per g of extract. Total soluble solids (TSS) concentration of the *P. ostreatus* fruiting bodies was measured by placing a small amount of sample on the digital refractometer and reading was recorded in Brix unit. The total sugar concentration in the mushroom fruit bodies sample was obtained by anthrone method as reported by [28].

#### Determination of K, P, and N contents

Fruiting bodies of *P. ostreatus* were collected and dried in an oven at 60 °C for 48 h to ensure complete moisture removal. The dried samples were then ground to a fine powder and passed through a 1-mm sieve. An aliquot of 1 g of the ground sample was subjected to digestion in 0.6 mol/L nitric acid (HNO₃) to prepare the sample solution. For the determination of potassium (K), the digested solution was analyzed using a flame photometer, following standard protocols. Phosphorus (P) content was quantified using the molybdenum blue method [29], a reliable spectrophotometric technique. Mushroom samples were first digested with sulfuric and perchloric acids to release phosphorus. The digested sample was then reacted with ammonium molybdate in an acidic medium, forming phosphomolybdic acid. This was reduced to molybdenum blue using a reducing agent, and the intensity of the blue color was measured at 880 nm using a spectrophotometer. A calibration curve with phosphorus standards was used to ensure accurate quantification. Nitrogen (N) content was determined by the Kjeldahl method, a well-established technique for total nitrogen analysis in organic materials [30].

#### Determination of vitamin c content

Ascorbic acid (vitamin C) content was measured quantitatively using a titration method with 2,6-dichlorophenolindophenol (DCPIP) dye, also known as Tillman’s reagent [31]. The titration process involved adding the DCPIP solution to the sample until a stable color change was observed, indicating the endpoint of the titration. The amount of DCPIP required was used to calculate the ascorbic acid content in the mushroom samples.

#### Enzyme activity assays

Peroxidase activities, including total peroxidase (total POD), manganese-dependent peroxidases (MnP), and manganese-independent peroxidases (MiP), were assessed using the procedure described by Martínez et al. [32] The assay employed 3-Methyl-2-benzothiazolinone hydrazone hydrochloride (MBTH, Fluka) as the substrate, which, in the presence of the enzyme, hydrogen peroxide (H₂O₂), and manganese (Mn), reacts with 3-dimethylaminobenzoic acid (DMAB, Aldrich) to produce a purple color. The reaction was conducted at a temperature of 30 °C and monitored at 590 nm using a spectrophotometer. The molar extinction coefficient for the MBTH/DMAB oxidation product was 32,900 M⁻¹ cm⁻¹. Activity of aryl-alcohol oxidase (AAO) was determined using veratryl alcohol (3,4-dimethoxybenzyl alcohol) as the substrate, based on the method described by Gutiérrez et al. [33]. The reaction conditions and spectrophotometric measurements were adjusted as per their procedure to accurately quantify AAO activity.

Polyphenol oxidase (PPO) was extracted from 20 g of mushroom tissue by homogenizing it in 100 mL of 100 mM phosphate buffer (pH 7.0) containing 10 mM ascorbic acid and 1% (w/w) polyethylene glycol. The homogenate was filtered through muslin cloth, and the filtrate was then centrifuged at 10,000 g for 20 min at 4 °C. The resulting supernatant was used for the PPO activity assay. PPO activity was measured using catechol as a substrate, following a spectrophotometric method as described by [34]. The increase in absorbance at 420 nm was monitored for 5 min. One unit of enzyme activity was defined as the amount of enzyme that produced a 0.001 change in absorbance per minute.

### Data analysis

Data was subjected to one-way analysis of variance (ANOVA) using SAS 9.4 software. Differences between treatment means were evaluated for statistical significance using Tukey’s HSD test at a significance level of α = 0.05. The data was analyzed using various statistical and visualization techniques. Heatmaps were created with the *pheatmap* package to identify patterns in the data. Radar plots generated with the *fmsb* package enabled comparison of multiple variables simultaneously. Bar plots and scatter plots produced with ggplot2 displayed summary statistics and relationships between variables. Principal component analysis (PCA) was performed using the *factoextra* and *FactoMineR* packages to uncover the primary sources of variation in the dataset.

## Results

### Analysis of variance (ANOVA) revealing seasonal, treatment, and interaction effects on *p. ostreatus* fruiting body traits

The ANOVA results showed significant effects of season, treatment, and their interaction on 11 traits measured for *P. ostreatus* fruiting bodies (Table 2). Seasons had a significant effect (*p* < 0.05) on all the studied attributes, including soluble sugar content (SSC), fruiting bodies yield (FBY), potassium (K), total phenolic content (TPC), polyphenol oxidase (PPO), spent weight (SW), and biological efficiency (BE), while vitamin C (VC), nitrogen (N), phosphorous (P), and peroxidase (POD), revealed non-significant variations. Treatment significantly affected (*p* < 0.05) the mean squares of all the studied traits. Furthermore, the interaction between season and treatment was significant (*p* < 0.05) for all traits except for TPC, VC, P, and POD. While both season and treatment influenced several mushroom traits on their own, their combined interaction highlights how one factor modified the impact of the other for some quality measures according to the ANOVA mean squares.

**Table 2:** Analysis of variance (ANOVA) showing the effects of season, treatment, and their interaction on studied parameters under potassium humate and wheat straw rate (RWS) treatments

Source	DF	SSC	FBY	VC	*N*	K	*P*
**Season (S)**	1	0.040***	26.042***	0.565 ns	1.027 ns	11.927**	1.986 ns
**Treatments (T)**	6	1.306***	2147.186***	186.859***	622.375***	168.685***	1032.418***
S*T	6	0.001**	5.506***	0.271 ns	43.273***	4.342*	49.692 ns
**Error**	27	0.000	0.075	0.701	0.280	1.408	40.319
**Source**	**DF**	**TPC**	**POD**	**PPO**	**Spent**	**BE**	
**Season (S)**	1	0.122**	0.000 ns	114.985***	47.939***	26.931***	
**Treatments (T)**	6	1.138***	0.002***	523.527***	5037.985***	1337.542***	
S*T	6	0.009 ns	0.000 ns	26.801***	40.994***	14.563***	
**Error**	27	0.010	0.000	0.241	0.250	0.028	

** and *** indicated significance at *p* < 0.01 and *p* < 0.001), while "ns" indicate non-significant difference. SSC (Soluble Sugar Content), FBY (Fruiting Bodies Yield), BE (Biological Efficiency), K (Potassium), N (Nitrogen), P (Phosphorus), POD (Peroxidase Activity), PPO (Polyphenol Oxidase Activity), SW (Spent Weight), TPC (Total Phenolic Content), VC (Vitamin C)

### Comparative effects of seven treatment combinations on parameters studied across two growing seasons

**Fig. 1:**
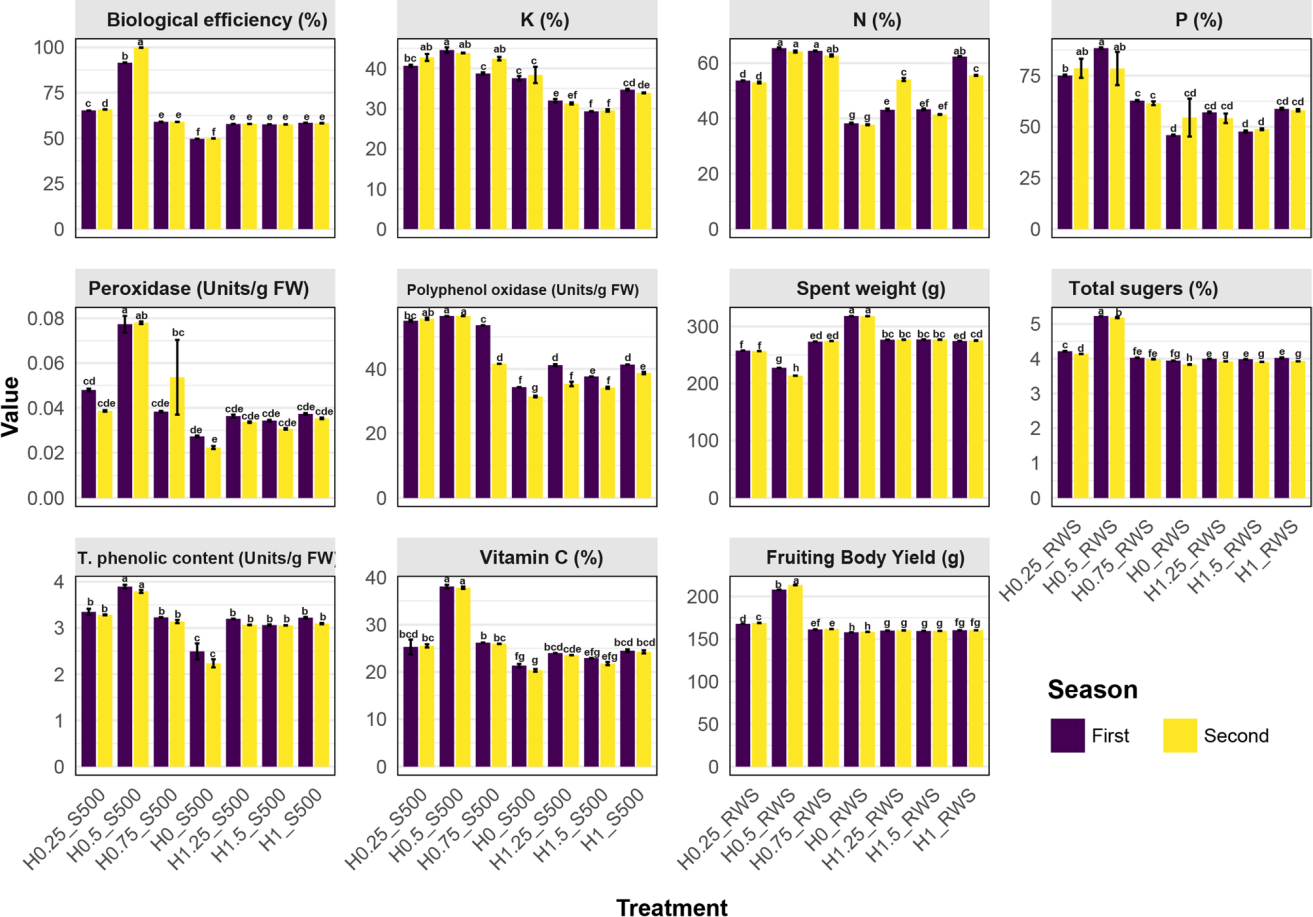
Comparison of different traits measured under various potassium humate and rate wheat straw treatment combinations across the two seasons of the study. Data are presented as means ± SE (standard error), with letters indicating statistically significant differences between treatments as determined by post-hoc multiple comparisons (*p* < 0.05). Purple bars represent the First season, while yellow bars represent the Second season. H0_RWS: 500 g of raw wheat straw (RWS) without potassium humate, H0.25_RWS: 500 g of RWS with 0.25 g of potassium humate, H0.5_RWS: 500 g of RWS with 0.5 g of potassium humate, H0.75_RWS: 500 g of RWS with 0.75 g of potassium humate, H1_RWS: 500 g of RWS with 1 g of potassium humate, H1.25_RWS: 500 g of RWS with 1.25 g of potassium humate, H1.50_RWS: 500 g of RWS with 1.5 g of potassium humate

The mean comparison of the influence of seven treatment combinations (H0_RWS, H0.25_RWS, H0.50_RWS, H0.75_RWS, H1_RWS, H1.25_RWS, and H1.5_RWS) across the two years showed notable variations across traits (Table 2). Overall, H0.50_RWS demonstrated superior performance across most of the traits compared to the other treatments, making it the most influential combination for optimizing plant performance across the two years (Fig. 1). For SSC, treatment H0.50_RWS consistently produced the highest values (5.23 and 5.19 mg/g FW) across both years. Fruiting bodies yield also peaked under H0.50_RWS in both years, with the highest values (208.06 and 213.47 g). Similarly, H0.50_RWS demonstrated superior results for VC (38.051 and 37.790 mg/100 g FW), indicating enhanced quality performance compared to the other treatments. Regarding the concentrations of the nutrient N, P, and K, the treatment H0.50_RWS yielded the highest levels of N (65.37 and 64.17 mg/100 g DW), K (44.57 and 43.83 mg/100 g DW), and P (88.44 and 78.56 mg/100 g DW), suggesting a positive impact of this treatment on nutrient uptake.

The treatment H0.50_RWS showed elevated TPC at 3.90 and 3.79 mg/100 g FW, along with the highest levels of PPO, recorded at 56.43 and 56.45 units/mg protein, in the first and second seasons, respectively. Peroxidase The levels of POD were consistent at 0.08 units for both seasons. Additionally, biological efficiency was maximized under H0.50_RWS, reaching 91.51% and 99.88% across the two seasons. In contrast to all treatment and traits studied, the spent weight values were the highest in H0_RWS for both years (318.14 and 317.99 g).

### Principal component analysis of treatment effects on *P. ostreatus* on yield, quality, and biochemical traits

**Fig. 2:**
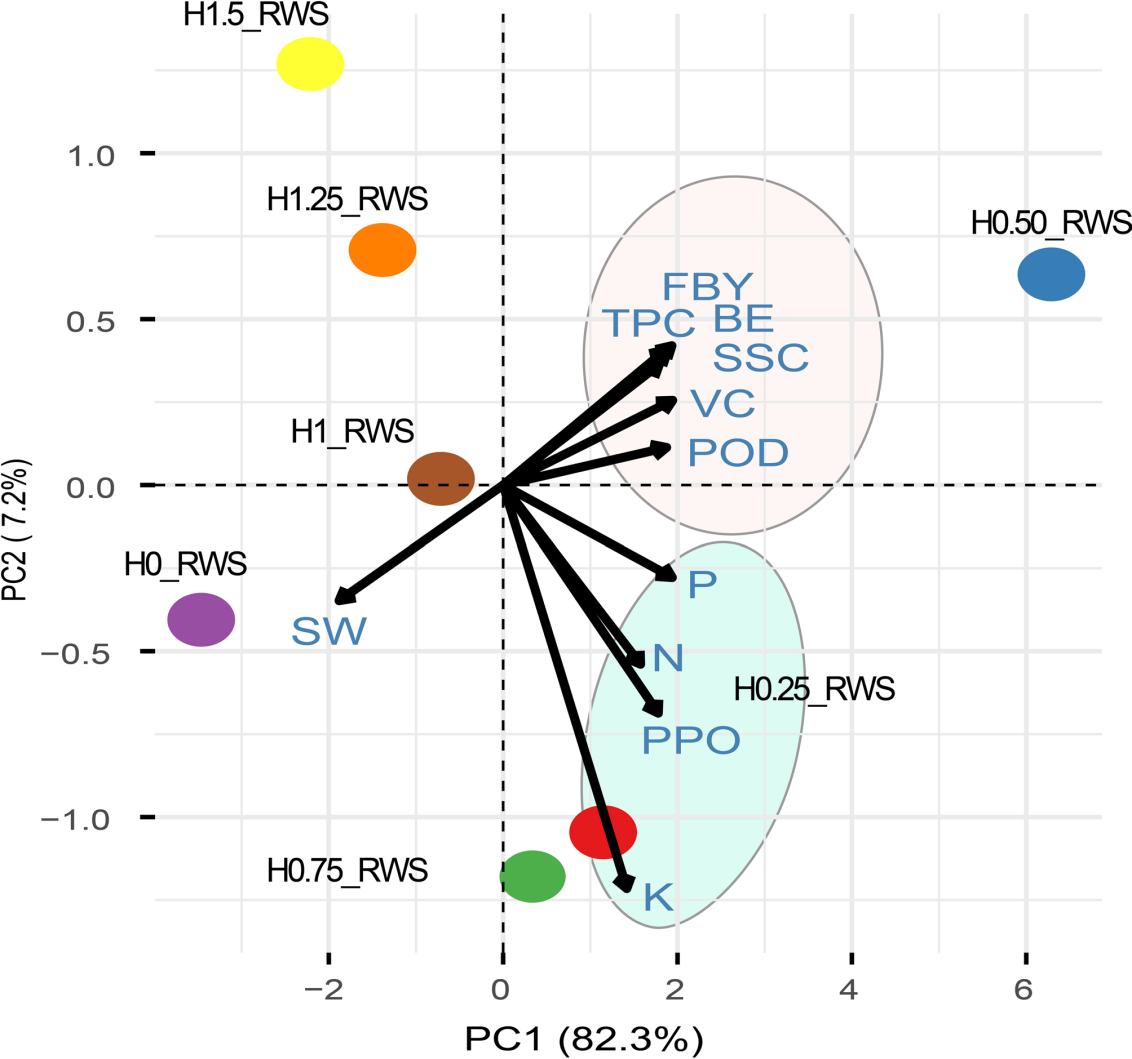
Principal component analysis (PCA) of eleven measured traits under seven treatment combinations varying in potassium humate concentration and raw wheat quantity. PC1 and PC2 showed explained variation of 82.3% and 7.2%, respectively

The Principal Component Analysis (PCA) biplot revealed intricate relationships between the measured traits and their responses to various potassium humate with RWS treatments (Fig. 2). The first two principal components accounted for a substantial 89.5% of the total variance, with PC1 and PC2 explaining 82.3% and 7.2%, respectively, indicating a robust representation of data variability. Trait associations demonstrated clear patterns within the biplot. Traits including FBY, TPC, BE, SSC, VC, and POD clustered tightly and showed strong positive correlations with PC1. This grouping indicates that these traits exhibited similar responses to the treatments and are likely interrelated in their impact on *P. ostreatus* productivity and quality. In contrast, SW exhibits a strong negative association with PC1, indicating a potential trade-off with a such yield-related parameter. Nutrient-related traits displayed diverse distributions, as N, P, K, and PPO clustered together with a slight negative association with PC2. Treatment effects were distinctly differentiated across the biplot. The H0.50_RWS treatment, positioned far right on PC1, showed the strongest positive association with yield and related quality parameters (FBY, TPC, BE, SSC, VC, and POD). Higher potassium humate concentrations (H1.25_RWS and H1.5_RWS) were in the upper left quadrant. The control treatment (H0_RWS) appeared in the lower left quadrant, emphasizing the overall positive impact of potassium humate application. The treatment H0_RWS was positioned very close to SW, indicating it as the most effective treatment for this yield trait. In contrast, H1_RWS was located near the origin, suggesting a relatively neutral effect compared to the other treatments.

### Impact of potassium humate and raw wheat straw extract treatments on nutritional, yield, and biochemical attributes of crops

**Fig. 3:**
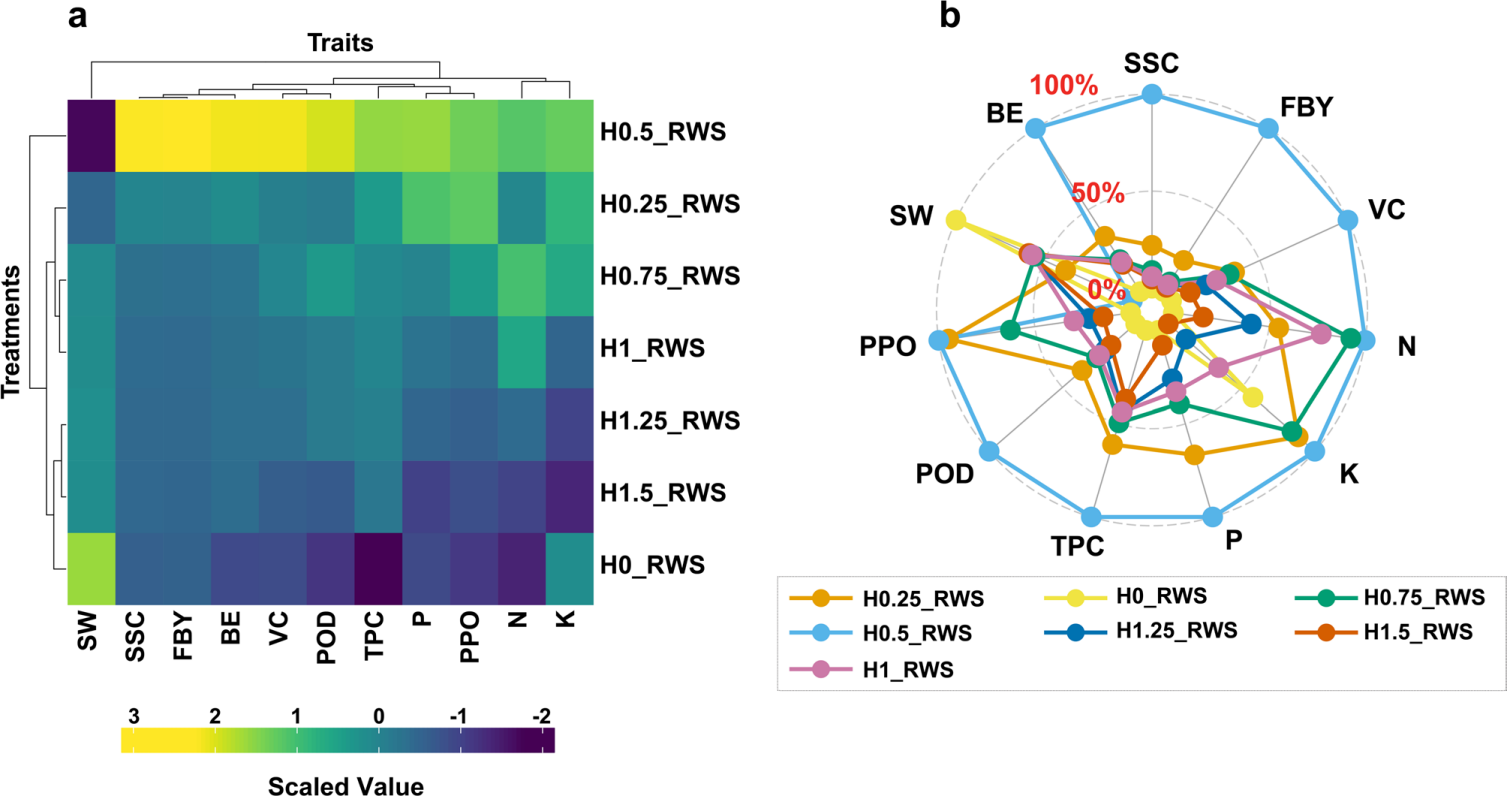
Trait responses of *P. ostreatus* to different treatments levels represented by heatmap and radar plot. **a** The heatmap illustrates scaled values of yield, quality and biochemical parameters across various treatments (H0_RWS to H1.5_RWS). Warmer colors (yellow to light green) indicate higher positive trait values, while cooler colors (dark blue to purple) represent lower values. **b** The radar plot compares the relative values of the same traits across treatments, with 0%, 50%, and 100% thresholds indicated by dashed lines. Fruiting bodies yield (FBY), soluble sugar content (SSC), biological efficiency (BE), potassium (K), nitrogen (N), phosphorus (P), peroxidase activity (POD), polyphenol oxidase activity (PPO), spent weight (SW), soluble sugar content (SSC), total phenolic content (TPC), and vitamin C (VC)

The heatmap and radar plot analyses (Fig. 3) provided a detailed evaluation of the effects of varying concentrations of potassium humate and raw wheat straw (RWS) extract on key nutritional, yield, and biochemical attributes of the *P. ostreatus*. The heatmap (Fig. 3a) illustrates distinct patterns in trait performance across the different treatment groups, ranging from the control (H0_RWS) to the highest dose (H1.5_RWS). Treatment H0.50_RWS, which corresponds to a moderate dose of potassium humate and RWS extract, exhibited the highest positive impact on multiple traits, particularly on SSC, FBY, BE, VC, and essential nutrients such as N and K. The prevalence of warmer colors in the heatmap (yellow to light green) for this treatment suggests that it produced the most favorable conditions for enhancing the nutritional and biochemical profiles of the fruiting body.

In contrast, the control treatment (H0_RWS), which did not receive potassium humate, showed the lowest values for several important traits, including yield, BE, VC, POD, and PPO. However, the control treatment exhibited the highest value for spent mushroom substrate. Lower treatment levels, such as H0.25_RWS, displayed moderate increases in trait values compared to the control, with a positive but less pronounced effect on nutritional and yield attributes. Treatment H0.75_RWS showed relatively higher values across most traits. Collectively, as the concentration of potassium humate and RWS extract increased to higher levels (H1_RWS, H1.25_RWS, and H1.5_RWS), a gradual decline in trait performance was observed. Specifically, the highest treatment level (H1.5_RWS) exhibited a significant reduction in values for key traits, including a marked decrease in potassium content. Additionally, P content exhibited a unique trend, with peak values observed in the mid-range treatments (H0.25_RWS to H0.75_RWS), followed by a decline at both the control and the highest supplementation levels. The radar plot (Fig. 3b) further visualized and confirmed these results, highlighting the relative performance of each treatment based on key attributes. The dashed lines in the radar plot represented threshold levels of 0%, 50%, and 100% performance for the measured traits. The H0.50_RWS treatment demonstrated the most balanced and optimal response, reaching the 100% threshold for several traits, including SSC and BE. This treatment clearly outperformed others in terms of nutrient accumulation and yield. In contrast, higher treatment levels such as H1.5_RWS often fell below the 50% threshold for key traits like K and VC. Lower supplementation levels, such as H0.25_RWS, also performed well, though they typically reached the 50% threshold rather than the 100% mark. Interestingly, treatments that showed high yield and nutrient content tended to correspond with lower values for the SW of mushroom.

### Correlation analysis reveals physiological and biochemical determinants of wheat yield

**Fig. 4:**
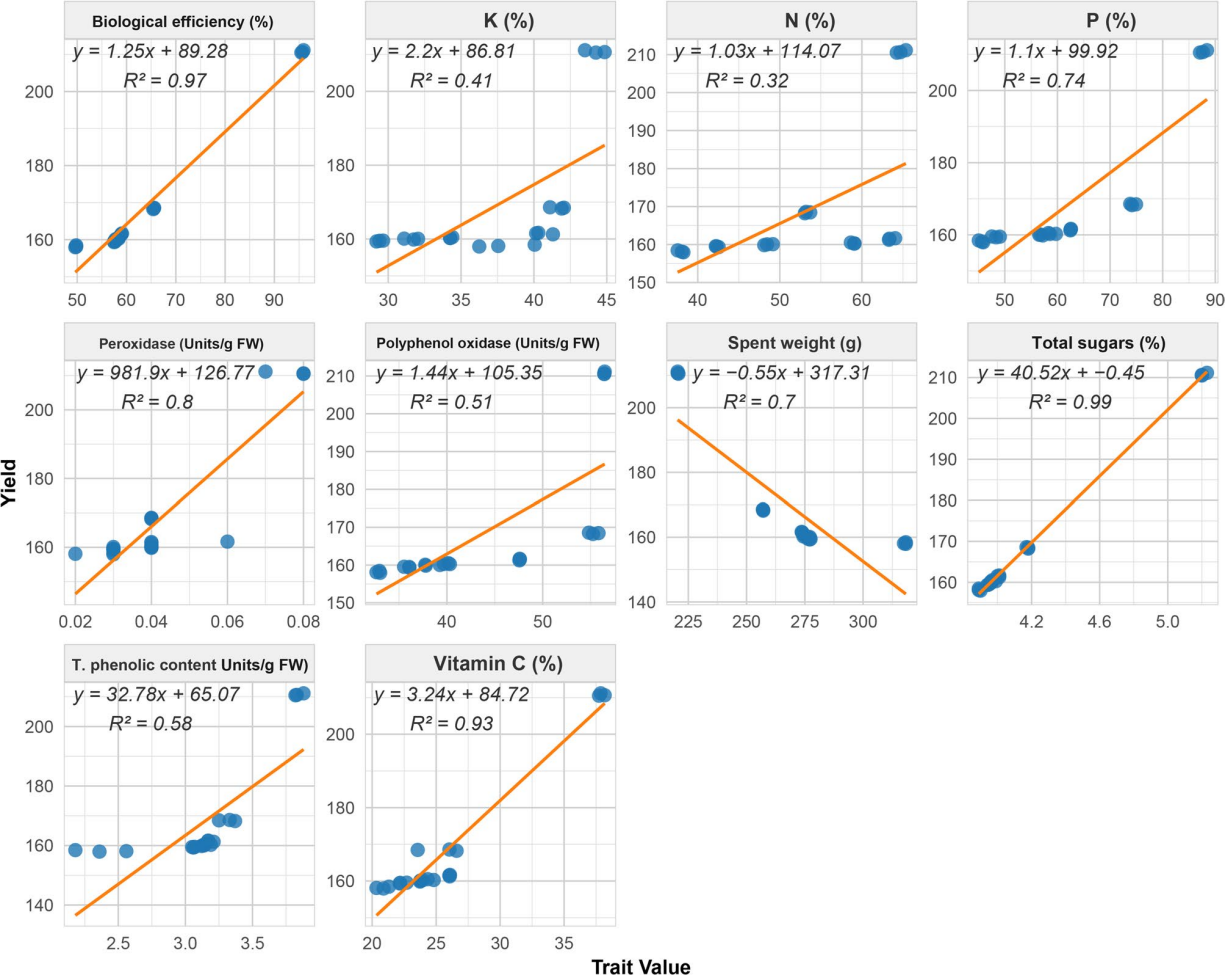
Scatter plots illustrating the relationships between various yield, nutritive and biochemical traits of *P. ostreatus*. Each panel represents a different trait plotted against yield, with linear regression lines and corresponding R² values. Soluble sugar content (SSC), biological efficiency (BE), potassium (K), nitrogen (N), phosphorus (P), peroxidase activity (POD), polyphenol oxidase activity (PPO), spent weight (SW), soluble sugar content (SSC), total phenolic content (TPC), and vitamin C (VC). The x-axis represents the trait value, while the y-axis shows yield in all panels

The BE demonstrated the strongest positive correlation with yield (R² = 0.97), suggesting a robust association between BE and crop productivity (Fig. 4). Similarly, VC showed a very strong positive relationship with yield (R² = 0.93). The SSC showed a highly strong positive correlation (R² = 0.99). Also, moderate positive correlations with yield were found with each of TPC and PPO activity (R² = 0.58 and 0.51, respectively). A strong positive correlation was observed between yield and P (R² = 0.74), while K and N showed moderate positive relationships with yield (R² = 0.41 and R² = 0.32, respectively). For weak relationship between yield and other parameters, POD activity revealed a relatively weak positive correlation with yield (R² = 0.18). Also, SW exhibited a strong negative correlation with yield (R² = 0.7), suggesting that higher values of this parameter are associated with decreased yields. These correlations provide insights into the complex factors influencing *P. ostreatus* yield.

## Discussion

The ANOVA results provided valuable insights into the effects of both season and treatment on the growth and quality of *P. ostreatus*. Notably, several traits showed significant variations, highlighting the impact of these factors on mushroom performance. The season factor was found to have a significant effect on soluble sugar content SSC, FBY, K, TPC, PPO, SW, and BE, indicating that environmental conditions during different seasons may have a substantial role in driving the physiological responses *P. ostreatus* [35].These seasonal variations likely stem from temperature-dependent enzymatic activities that regulate substrate decomposition and metabolite synthesis [36]. During optimal temperature ranges, enhanced cellulase and hemicellulose activities facilitate more efficient breakdown of wheat straw lignocellulosic components, directly translating to improved fruiting body yield and biological efficiency [26]. The temperature sensitivity of polyphenol oxidase activity explains its seasonal variation, as this enzyme’s catalytic efficiency is highly dependent on thermal conditions that affect protein conformation and substrate binding affinity [37]. The lack of significant seasonal effects on VC, N, phosphorus, and POD suggests that these traits are relatively stable and less influenced by seasonal variations, which is advantageous for maintaining consistent nutritional quality across different cultivation periods [35]. Treatment effects were significant for all measured traits, reinforcing the use of potassium humate and wheat straw in optimizing yield and enhancing the nutritional profile of *P. ostreatus*. Similar findings have demonstrated that substrate supplementation and nutrient amendments can lead to improved growth parameters and biochemical content [38]. Interestingly, the interaction between season and treatment was significant for most traits, indicating that the combined effects of these factors can either amplify or mitigate the response of *P. ostreatus*. For example, the lack of interaction effects on TPC, VC, P, and POD suggests that these traits are influenced independently by season or treatment, while other traits may be more susceptible to synergistic effects [39]. The significant treatment × season interactions for most traits, including nitrogen and potassium, reveal complex regulatory networks where potassium humate effectiveness varies with environmental conditions. The significant interaction effects for N and K indicate that the response to potassium humate treatments differs markedly between seasons, suggesting that environmental factors such as temperature and humidity modulate the effectiveness of these amendments [40]. During favorable seasons, enhanced metabolic activity may increase the demand for these nutrients, making the supplementation more effective. Conversely, during less favorable conditions, the fungus may exhibit reduced responsiveness to nutrient amendments due to overall metabolic downregulation. This interaction likely occurs through differential effects on mycelial membrane permeability and ion transport systems under varying temperature and humidity conditions, affecting nutrient concentration in tissues and metabolic flux distribution [41].

The comparative analysis of the seven treatment combinations across two growing seasons highlights the significant impact of potassium humate and wheat straw amendments on the growth, yield, and quality of *P. ostreatus*. Notably, the treatment H0.50_RWS consistently outperformed the other combinations, particularly in traits such as SSC, FBY, and VC, emphasizing the effectiveness of moderate levels of potassium humate in enhancing both quantitative and qualitative traits. This superior performance is aligned with previous studies, which have reported the beneficial effects of humic substances on *P. ostreatus* physiology and biochemical properties [42]. The increased vitamin C levels in this treatment demonstrated the potential to enhance the antioxidant properties of *P. ostreatus*, thereby improving its nutritional value [43]. This outcome is corroborated with the findings of [30], who showed that substrate supplementation with humic substances improves the accumulation of vitamins and antioxidants in *P. ostreatus*. Moreover, the significantly higher nutrient uptake observed in the H0.50_RWS treatment, including N, P, and K, underscores the role of potassium humate in improving nutrient availability and absorption, which were crucial for growth and biochemical composition [44]. The enhanced nitrogen concentration under H0.50_RWS treatment indicates stimulated amino acid synthesis pathways, particularly those involving glutamine synthetase and glutamate dehydrogenase enzymes. Higher nitrogen concentration in tissue translates directly to increased protein synthesis, supporting both mycelial growth and fruiting body development [44]. The elevated phosphorus concentration reflects enhanced energy metabolism through improved ATP synthesis and phosphorylation processes essential for cellular growth and reproduction [29]. It is important to distinguish that these measurements represent nutrient concentrations within the fungal tissue (content per unit weight) rather than total nutrient uptake (content relative to total biomass). This indicates that nutrient-deficient substrates lead to increased spent biomass, as *P. ostreatus* face challenges in optimizing growth under suboptimal conditions [45].The remarkable vitamin C enhancement (38.05 mg/100 g FW) under optimal treatment suggests activation of ascorbic acid biosynthetic pathways, specifically upregulation of L-galactono-1,4-lactone dehydrogenase, the rate-limiting enzyme in fungal ascorbic acid synthesis [43]. This enhancement serves dual functions: strengthening antioxidant defense mechanisms against oxidative stress and supporting collagen-like structural protein synthesis in fruiting body cell walls [31].The elevated soluble sugar content indicates enhanced carbohydrate metabolism and storage capacity. Potassium humate likely stimulates glycogen phosphorylase and amylase activities, promoting efficient conversion of complex carbohydrates from wheat straw into readily available sugars for metabolic processes and structural development [46].

A comprehensive understanding of the intricate relationships between yield, quality, and biochemical traits of *P. ostreatus* under different potassium humate treatments with rice wheat straw was provided by PCA biplot in this study. The biplot utilized in this study elucidated not only the relationships between traits but also underscored the optimal treatment combinations for maximizing *P. ostreatus* production and biochemical enhancement, providing valuable insights for future cultivation strategies. The strong clustering of FBY, TPC, BE, SSC, VC, and POD along PC1 reflected their interconnected roles in enhancing the productivity and quality *P. ostreatus*, particularly under the H0.50_RWS treatment. This pattern aligned with previous studies that demonstrated how humic substances could positively influence the synthesis of bioactive compounds and improve yield parameters [47]. The negative association of SW with PC1 suggests a trade-off between yield and substrate decomposition, indicating that higher productivity may be linked to lower spent substrate weights. This finding is consistent with the idea that optimal substrate utilization maximizes yield efficiency [4]. In contrast, higher concentrations of potassium humate (H1.25_RWS and H1.5_RWS) showed a more neutral or slightly negative influence, possibly due to saturation effects, as has been previously noted in studies involving high levels of humic acid amendments [30]. The control treatment (H0_RWS) clustered closely with SW, highlighting its limited effectiveness in promoting yield and quality. In contrast, H1_RWS’s central position near the origin suggests a more balanced, though less pronounced, effect compared to the superior performance of H0.50_RWS.

The nuanced effects of potassium humate and wheat straw extract treatments on *P. ostreatus* performance were further underscored by heatmap and radar plot analyses. Overall, these analyses reinforce the critical role of potassium humate in crop performance while demonstrating that moderation in application is key to achieving balanced and optimal outcomes. The visualizations revealed clear differences in trait responses across treatment concentrations. Treatment H0.50_RWS consistently produced optimal outcomes across multiple parameters, as evidenced by favorable heatmap colors and peak radar plot scores. This treatment showed a balanced impact on nutritional properties, yield, and biochemical characteristics. These results established it as the most effective option, supporting previous research that showed moderate humate levels enhance nutrient absorption and crop quality. Reduced supplementation amounts, such as H0.25_RWS, demonstrated beneficial but less significant effects, generally enhancing characteristics without reaching the ideal threshold shown in the H0.50_RWS treatment. Meanwhile, the control (H0_RWS) consistently exhibited the lowest performance, highlighting the necessity of substrate enrichment for enhanced growth and nutrient accumulation. As the concentrations increased beyond the optimal level (H1_RWS to H1.5_RWS), a gradual decline in performance across several traits became evident, a common phenomenon associated with excessive humic supplementation. This pattern reflects findings in other studies where high levels of humic substances may inhibit nutrient uptake and overall crop performance [48]. Interestingly, the inverse relationship between yield and SW observed in higher treatment groups further suggests that nutrient optimization may come at the expense of substrate weight, likely due to more efficient resource use within the *P. ostreatus*.

The correlation analysis revealed critical insights into the physiological and biochemical factors that significantly influence *P. ostreatus* yield. These correlations provide a comprehensive understanding of the multifactorial nature of yield determination in *P. ostreatus* and offer valuable guidelines for improving both physiological and biochemical attributes to enhance productivity. The strong positive correlations between yield and traits such as BE, VC, and SSC underscore their pivotal roles in enhancing productivity. These findings align with previous research, where BE has been recognized as a key indicator of yield efficiency in mushroom cultivation [49]. Similarly, the robust associations of VC and SSC with yield suggest that biochemical enhancements, particularly in antioxidant content and sugar accumulation, may play a vital role in promoting growth [43, 50]. In contrast, the strong negative correlation between SW and yield suggests that heavier substrate weight could impede optimal *P. ostreatus* growth by limiting resource availability, a finding consistent with studies on substrate optimization [51]. The positive relationships with nutrient traits such as P, K, and N further highlight the importance of nutrient availability in driving yield, reinforcing the need for balanced substrate composition.

The identification of H0.50_RWS as the optimal treatment provides a practical framework for commercial mushroom production optimization. The concentration-response relationship demonstrates that precise nutrient management is more critical than maximum supplementation, suggesting that cost-effective production can be achieved through targeted rather than excessive input use [35]. The seasonal stability of key quality parameters (VC, N, P, POD) indicates that nutritional quality can be maintained consistently across production cycles, providing reliability for commercial operations. However, the seasonal variability in yield parameters suggests that production planning should account for environmental factors to optimize harvest timing and resource allocation [36]. The enzyme activity relationships provide diagnostic tools for production monitoring, where peroxidase and polyphenol oxidase activities can serve as indicators of substrate utilization efficiency and quality development, enabling real-time production optimization decisions [4].

## Conclusions

This study provides valuable insights into optimizing the cultivation of *P. ostreatus* using potassium humate and raw wheat straw treatments. Our comprehensive analysis across two growing seasons revealed that moderate levels of potassium humate, particularly the H0.50_RWS treatment, significantly enhanced multiple key attributes of *P. ostreatus* production. Notably, this treatment led to improvements in fruiting body yield, soluble sugar content, biological efficiency, and vitamin C levels. The study also highlighted the importance of balanced supplementation, as higher concentrations of potassium humate showed diminishing returns on several traits. The findings of PCA demonstrated strong positive associations between yield-related parameters and biochemical traits, suggesting a holistic improvement in mushroom quality and productivity under optimal treatment conditions. These findings have substantial implications for *P. ostreatus* cultivation practices, offering a potential pathway to enhance both yield and nutritional value simultaneously. Future research should focus on validating these results across diverse environmental conditions and over extended cultivation cycles. Additionally, investigating the molecular mechanisms underlying the observed improvements could provide deeper insights into fungal physiology and metabolism. Economic analyses to assess the cost-effectiveness of potassium humate supplementation in large-scale production systems would be crucial for practical implementation. Furthermore, exploring the potential impacts of these treatments on *P. ostreatus* shelf-life and post-harvest quality could add significant value to the supply chain.

## Data Availability

Data that support the findings of this study are available from the corresponding author upon reasonable request.
